# Social Information Processing in Substance Use Disorders: Insights From an Emotional Go-Nogo Task

**DOI:** 10.3389/fpsyt.2021.672488

**Published:** 2021-05-28

**Authors:** James M. Bjork, Lori Keyser-Marcus, Jasmin Vassileva, Tatiana Ramey, David C. Houghton, F. Gerard Moeller

**Affiliations:** ^1^Institute for Drug and Alcohol Studies, Virginia Commonwealth University, Richmond, VA, United States; ^2^Division of Therapeutics and Medical Consequences, National Institute on Drug Abuse, Bethesda, MD, United States; ^3^Department of Psychiatry and Behavioral Sciences, Center for Addiction Research, University of Texas Medical Branch, Galveston, TX, United States

**Keywords:** impulsivity, social information processing, substance use disorder, cocaine, cannabis (marijuana), opioids, go nogo task

## Abstract

Positive social connections are crucial for recovery from Substance Use Disorder (SUD). Of interest is understanding potential social information processing (SIP) mediators of this effect. To explore whether persons with different SUD show idiosyncratic biases toward social signals, we administered an emotional go-nogo task (EGNG) to 31 individuals with Cocaine Use Disorder (CoUD), 31 with Cannabis Use Disorder (CaUD), 79 with Opioid Use Disorder (OUD), and 58 controls. Participants were instructed to respond to emotional faces (Fear/Happy) but withhold responses to expressionless faces in two task blocks, with the reverse instruction in the other two blocks. Emotional faces as non-targets elicited more “false alarm” (FA) commission errors as a main effect. Groups did not differ in overall rates of hits (correct responses to target faces), but participants with CaUD and CoUD showed reduced rates of hits (relative to controls) when expressionless faces were targets. OUD participants had worse hit rates [and slower reaction times (RT)] when fearful faces (but not happy faces) were targets. CaUD participants were most affected by instruction effects (respond/“go” vs withhold response/“no-go” to emotional face) on discriminability statistic A. Participants were faster to respond to happy face targets than to expressionless faces. However, this pattern was reversed in fearful face blocks in OUD and CoUD participants. This experiment replicated previous findings of the greater salience of expressive face images, and extends this finding to SUD, where persons with CaUD may show even greater bias toward emotional faces. Conversely, OUD participants showed idiosyncratic behavior in response to fearful faces suggestive of increased attentional disruption by fear. These data suggest a mechanism by which positive social signals may contribute to recovery.

## Introduction

In light of the staggering worldwide economic and psychosocial toll of substance use disorder (SUD) (1), translating knowledge on mechanisms of addiction into treatments is critical. However, SUD treatments have proven elusive (2), with few approved pharmacotherapies. One possible reason for this failure in translation may be neglect of the *social* element of compulsive drug use (3). As fundamentally social animals, humans must properly interpret social information to properly calibrate behavioral responses (4), such as correcting transgressive behavior. Extensive psychosocial research indicates that deficits in social function may both contribute to and result from SUD [reviewed in (5)]. Conversely, robust social integration with peers has been shown to prevent onset of problematic substance use in both humans (6) and rodents (7). Moreover, positive social interactions (supports) have been shown to be critical for SUD treatment retention (8, 9).

Research on social functioning in SUD has extensively documented reduced connectedness to others, where much of this alienation stems from parasitic behavior (using and exploiting others) and other social consequences of compulsive use. However, this alienation (and impaired capacity for reconciliation) may also stem from aberrations in social information processing (SIP) that can occur at different stages, ranging from faulty initial perception of emotion in others to impaired “theory of mind” (ToM) inference of the mental states and emotions of others. Impairments in early-stage emotion recognition expressed in face images [such as with intermediate facial “morphs,” reviewed in (10)] have been detected in abusers of alcohol (11), cocaine (12, 13), cannabis (14, 15), opiates (16), and poly-substances (17).

ToM deficits have also been documented in different SUDs and have been linked to impaired functional outcomes (18). For example, using paradigms that involve ratings of audiovisual vignettes, impaired ability to accurately perceive the mental states or perspectives of others (affective ToM) has been found in heavy users or abusers of alcohol (19), cocaine (20), cannabis (21), opiates (22, 23), and poly-substances (24). During SUD recovery, impaired interpretation of emotion would potentially limit the capacity of social reinforcement (either positive or negative) to discourage substance abuse. Indeed, the ability to differentiate between negative emotional states has been shown to protect against initial lapses following SUD treatment (25).

Virtually unexplored, however, are abnormalities in initial *attentional capture* by (bias toward) emotional social information (5). Early attentional capture by emotional social cues plays an important role in all other downstream aspects of SIP, wherein vigilance toward early signs of emotional information enables adaptive responding and self-regulation. Facial stimuli are thought to capture attention via “bottom-up” processes in individuals to whom they are motivationally salient (26), and so may impede performance on cognitive tasks by competing for limited cognitive resources. Accordingly, individuals with social anxiety disorder showed relatively increased RT effects of task-irrelevant faces, with sustained RT interference and enhanced anterior insula (salience network) recruitment by angry faces (27). Indeed, a selective early attentional bias for certain social emotional cues (e.g., fear or anger) can be maladaptive, reflecting hypervigilance and inflexible behavioral response styles. For example, attentional bias toward negatively-valenced emotional faces in depressive and anxiety disorders is believed to reflect poor self-esteem and hypervigilance for social threat-related cues (26). This hypervigilance for social threat is then believed to facilitate attributional bias (i.e., a tendency to ascribe negative intentions in others) and socially avoidant behavior patterns. In sum, due of appreciable incidence of anxiety and depression in SUD, a potential exists for negative faces to also capture attention in some SUD due to anxiety and depression symptomatology.

In terms of behavioral readouts, attentional capture by positive or negative social information in different SUD may be reflected in reaction times (RT), which might be shorter if the stimuli are conditioned, simple, and appetitive (approach), or prolonged if complex or aversive (avoidance). For example, in food-deprived participants, use of food images as nogo (non-target) stimuli in a go-nogo task increased “false alarm” (FA) commission error responses (28). This effect was not apparent when participants were sated. Relatedly, some cognitive training approaches (e.g., for overeating) have attempted to *manipulate* implicit approach bias by having participants perform go-nogo tasks wherein desired reward (e.g., chocolate bar) images are assigned to be “nogo” stimuli [reviewed in (29)].

Existing research on early attentional SIP biases in SUDs, however, has revealed mixed findings. Persons who use high levels of cannabis have demonstrated reduced attentional capture by fearful faces (30), suggesting either a signature or mechanism of the extended anxiolytic properties of cannabis. Conversely, a functional neuroimaging study on persons with alcohol dependence found increased recruitment of the salience network by angry faces (31). These discrepant findings could be the result of differences between users of different substances in their SIP proclivities, perhaps as a signature of substance-specific alterations of salience or motivational networks.

Personality traits associated with addiction to different substances may offer an account of substance-specific proclivities. For example, in a twin study, cannabis use disorder was more strongly linked to novelty-seeking, whereas sedative (e.g., heroin) use was more linked to neuroticism (32). These findings were also reflected in other surveys wherein cannabis abuse was linked to increased openness (33, 34) and opioid abuse to elevated neuroticism (34). More recently, machine-learning analysis of laboratory behaviors and self-reported features in SUD identified mood disturbance and elevated interpersonal/affective psychopathy factor scores (but not impulsive/antisocial psychopathy factor scores) as primary distinguishing features of OUD compared to stimulant use disorders (35). In contrast, sensation-seeking was a unique and strong predictor of stimulant use disorder (35). Relatedly, OUD and opiate use (and preference) is more commonly linked to use in socially-withdrawn “home” environments, whereas stimulant use (and preference) is more linked to use in novel environments (36). These preferences have been instantiated in differential activation of reward circuitry in humans during rumination about substance use in these concordant vs. discordant social contexts (37). This social context selectivity has been attributed to the physiological conformity between the depressive effects of opiates in safe and subdued “resident” contexts vs. the arousing and alerting effects of stimulants that would be adaptive in novel environments with potentially-dangerous conspecifics (38, 39).

The aforementioned findings suggest a possibility for greater attentional capture or approach behavior toward positively-valenced (happy) faces in persons with cannabis use disorder (CaUD) and greater attentional capture by or aversion to negatively-valenced (sad/angry/fearful) faces in persons with opioid use disorder (OUD). To our knowledge, no experiment has probed attentional bias toward affective faces in multiple SUD populations. Further, no experiments have examined the potential for the salience of emotional faces to disrupt behavior inhibition specifically. This could be especially important for SUD in light of the increased motoric impulsivity characteristic of addiction to most substances of abuse (40). To investigate this, we administered an emotional go-nogo (EGNG) task (41) to individuals with cocaine use disorder (CoUD), cannabis use disorder (CaUD), opioid use disorder (OUD), and neurotypical controls. In the EGNG task, participants are instructed in some task blocks to respond to faces expressing a certain emotion (in our version fear or happiness), but withhold responses to expressionless faces. In other blocks, the reverse instruction applies.

Although a paucity of extant literature makes hypotheses tenuous, we nevertheless anticipated two main effects. First, we expected to replicate the primary Tottenham et al. (41) finding of an “instruction” effect, wherein FA rates would be higher (and thus overall signal detection accuracy would be lower) for all groups when they were instructed to “no-go” (not respond) to emotional faces relative to when they were “go” (target) stimuli. We also expected that in line with findings across tasks that appetitive salient stimuli elicit more robust motor approach behavior [e.g., (42)], we expected to find faster responses and more hits to happy face targets. With respect to differences with SUD, we envisioned opposing possibilities. Among SUD, CoUD in particular is most robustly linked to deficits in executive function (EF) (36, 40, 43), which includes behavioral inhibition as a core component (44). Not only is the EGNG itself an impulsivity task, but impaired EF has been shown to exacerbate facial attentional bias (45, 46). In this light, CoUD participants may show a vulnerability to respond to emotional faces as a result of their already-elevated impulsivity. Thus, one could expect that the aforementioned normative effects of salient emotion images in controls would be larger (i.e., instruction X group interaction) in persons with CoUD. Based on the linkages between CaUD and novelty-seeking, we also expected the CaUD participants might show exaggerated instruction effects as well. Alternatively, in light of findings (mentioned above) that decrements in detection of emotions in facial photographs themselves have been found in several SUD, we might find instead a blunted effect of instruction (respond to emotion vs. withhold to emotion) in SUD groups relative to the control group, due to inability to *detect* the emotion in some or all emotion-valenced images. Finally, since facial-emotion attentional bias is most established with affective disorders (26), in light of the strong role of negative emotion in OUD even relative to other SUD (35), we expected aversive attentional capture by fearful faces in particular would likely be greater in OUD, to in turn prolong RT and impair task performance in blocks involving fearful faces. In light of the Torrence et al. (30) finding of blunted evoked potential responses to fearful faces in cannabis users, a reasonable expectation would be a blunted fearful-face interference (RT) effect in the CaUD participants.

## Materials and Methods

All recruitment and testing procedures were reviewed and approved by the Institutional Review Board of Virginia Commonwealth University (VCU).

### Participants

Participants were adults age 25–70, and were a mix of persons recruited from Richmond, Virginia-area SUD treatment facilities, persons with SUD not in treatment as well as neurotypical controls recruited from the community using electronic and print media and flyers. All participants were recruited for a feasibility study of a Phenotypic Assessment Battery (PhAB; which included the EGNG task) commissioned by the National Institute on Drug Abuse (NIDA) for potential standardized use in NIDA-funded clinical trials (47). Respondents first underwent a telephone pre-screen, where likely-eligible individuals were invited to in-person screening. Inclusion criteria centered on meeting DSM-5 criteria for moderate or severe CaUD, CoUD, or OUD, per psychiatric interview (below). Participants who met criteria for exactly one of CaUD, CoUD or OUD moderate-severe were placed into the respective substance group. Participants who met criteria for more than one of these SUD (moderate-severe) were assigned to the substance group of greatest DSM-5 severity. Participants who met identical severity of more than one substance were assigned based on their preferred substance. Exclusion criteria were history of seizures (excluding childhood febrile seizures), or loss of consciousness from traumatic injury for more than 30 min, current psychosis, mania, or suicidal/homicidal ideation, current DSM-5 diagnosis of any psychoactive substance use disorder other than opioids, marijuana, stimulants, or nicotine. Diagnosis of mild to moderate AUD was not exclusionary. Controls were defined by absence of current regular substance use and failure to meet criteria for any past-year SUD. After exclusion of additional participants whose data indicated misinterpretation of task instructions in at least one task block (see below), the analyses herein include 31 individuals with Cocaine Use Disorder (CoUD), 31 individuals with Cannabis Use Disorder (CaUD), 79 individuals with Opioid Use Disorder (OUD), and 58 healthy controls. Demographic, psychiatric and substance use characteristics of participants are shown in Table 1.

**Table 1:** Participant characteristics.

		**Controls**	**CoUD**	**CaUD**	**OUD**	***F*/Chi-sq**	***P***
Sex		25 M, 33 F	20 M, 11 F	19 M, 12 F	44 M, 34 F	4.984	0.173
Age		43.5a (12.9)	53.6b (7.3)	40.3a (10.2)	43.6a (10.4)	9.483	<0.0001
	Range	25–70	33–66	25–62	26–69		
Shipley (verbal) AQ standard score		94.5a (17.4)	80.3b (14.3)	82.0b (15.2)	86.9b (13.9)	6.609	<0.001
	Range	34–125	55–105	47–104	50–116		
Shipley (non-verbal) BQ standard score		98.0 (16.2)	94.7 (9.3)	95.4 (11.8)	94.7 (10.8)	0.766	0.514
	Range	65–129	81–119	75–117	69–127		
Comorbid CoUD (%)		-	-	10 (31.3%)	37 (47.4%)		
Cocaine-positive urine (%)		0	22 (71%)	6 (19.4%)	21 (26.9%)		
Comorbid CaUD (%)		-	12 (39%)	-	19 (24%)		
Cannabis-positive urine (%)		2 (3.4%)	8 (25.8%)	24 (77.4%)	20 (25.3%)		
Comorbid OUD (%)		-	4 (12.9%)	3 (9.4%)	-		
Opioid-positive urine (%)		0	3 (9.7%)	2 (6.5%)	69 (87.3%)		
Comorbid AUD (%)			6 (19.4%)	14 (45.2%)	12 (15.4%)		
MDMA-positive urine (%)		0	0	0	0		
Mood/anxiety disorders (past)		8 (13.8%)	2 (6.5%)	3 (9.4%)	6 (7.7%)		
Mood/anxiety disorders (recurrent)		2 (3.4%)	0	2 (6.3%)	5 (6.4%)		

*a, b, different letters denote means that are significantly different*.

### Assessments

#### Phenotypic Assessments

Participants underwent the Mini International Neuropsychiatric Interview V 7.0.2 (MINI) (48) to determine SUD and other DSM-5 psychiatric disorders. A time-line follow-back interview (49) was used to clarify SUD. Recent substance use was corroborated by urine drug screen results. Past-week affective symptomatology was probed using the PROMIS Depression 4a and Anxiety 4a scales (50) as self-report measures. In addition, participants completed the computerized Shipley-2 scale (51). We include for illustrative purposes here its Composite-B (BQ) scale as a comparator metric of (fluid) IQ between groups that is thought to be less affected by task-irrelevant crystallized verbal skills.

#### Emotional Go-NoGo Task

This was a version of the original EGNG task of (41) with a few differences. First, only the fear and happy blocks were administered for brevity. Notably, these were the emotions most readily discriminated in neurotypical adults in the (41) study. Second, the original grayscale Ekman face stimuli were replaced by more contemporary color images of 12 actors (6 M, 6 F) of the Umeå University Database of Facial Expressions (52). Third, no-go stimuli were presented in 25% of trials instead of 30%.

**Figure 1:**
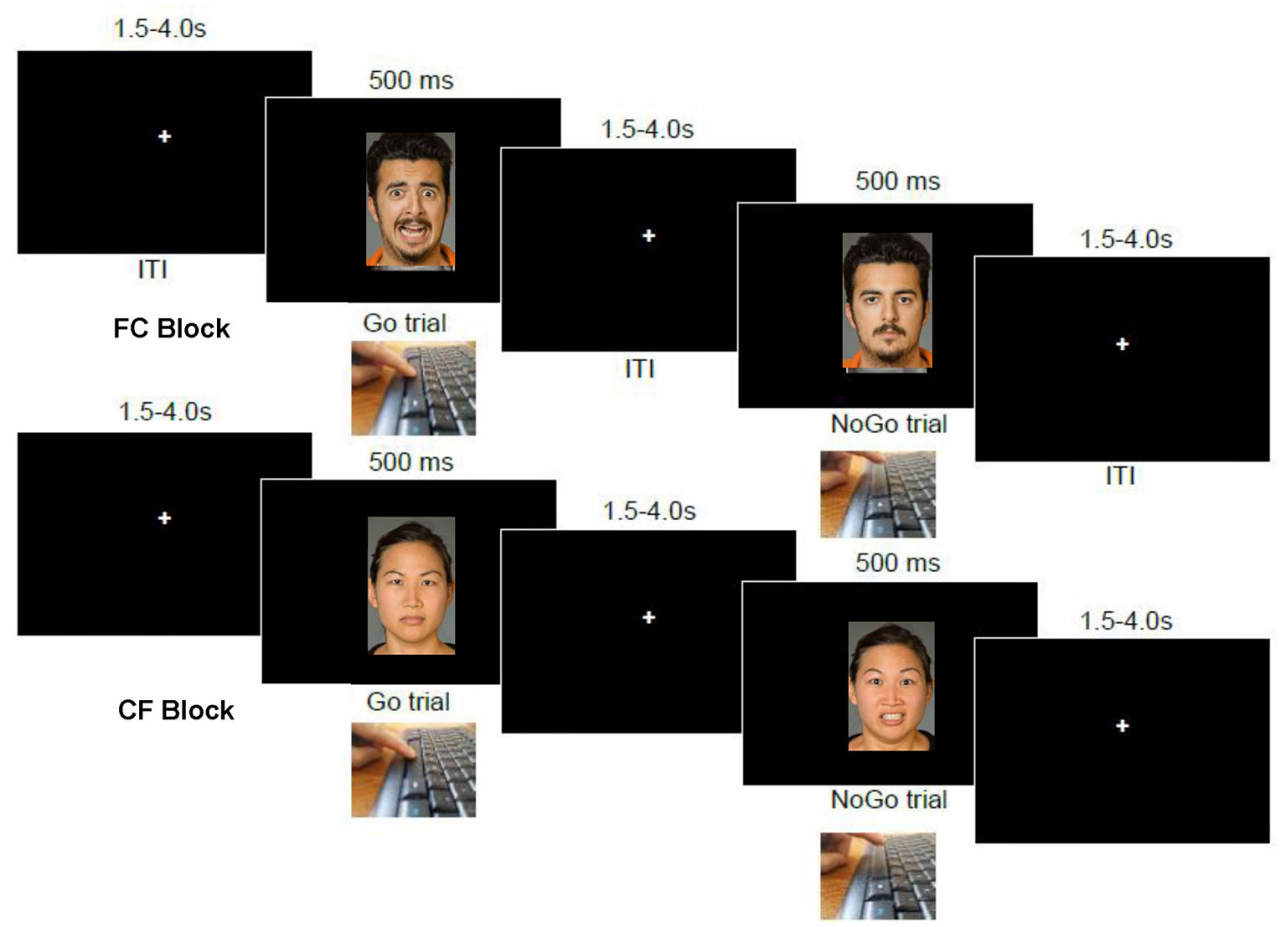
Emotional go-nogo task. At top is a sequence of events in an “FC” block, wherein participants are to respond on the space bar to fearful images but not to calm (expressionless) images. The bottom image series shows the reverse contingency in operation, “CF”, wherein participants are to instead withhold responses to fearful faces, and respond only to calm (expressionless) faces.

In each of four task blocks of 48 trials, the participant saw a face image presented in the middle of the screen for 500 ms followed by 1,000 ms fixation crosshair (see Figure 1). Responses were counted within this 1,500 ms window. Depending on the presence (or absence) of a specific emotion in the face image, the participant was instructed to respond using the space bar as quickly as possible. At the beginning of each block, the specific instruction for that block was displayed to the subject until a space bar press to proceed. In the HC block, the participant was instructed to respond to “happy” faces, but to withhold responses to “plain” (calm, expressionless) faces. In the CH block, this response contingency was reversed. Similarly, in the FC block, the participant was instructed to respond to “fearful” faces, but to withhold responses to calm faces, and the reverse contingency was instructed for the CF block. Importantly, requiring semantic evaluation of the *emotion conveyed* by emotional faces as the task instruction (vs responding based on gender or some other stimulus feature) has been shown to maximize emotion effects on behavior (53). The blocks were presented in four possible orders, which was randomly-determined for each task administration.

#### Data Analysis

For each of the HC, CH, FC, and CF blocks, rates of correct responses (hits to targets), commission errors [“false alarm” (FA) responses to non-targets] and median RT to targets (hits) were analyzed as the key dependent measures. Median values for RT were used to minimize outlier trial effects. RT was not analyzed for FA trials because several participants did not make any FA responses in at least one task block. We also calculated the omnibus signal detection statistics A and b (54). A is a non-parametric statistic of sensitivity (overall performance accuracy) akin to d-prime, but does not require substitution of values in performances with no errors. An A value of 1.0 indicates a perfect task performance. The metric b (log-transformed for normality) reflects response bias, where higher values indicate a more conservative responding strategy, and lower values a more liberal responding strategy.

We conducted repeated measures ANOVA on each of hit rates, false alarm rates, and RT to targets, with task instruction [instruction to respond to emotional faces (FC, HC blocks) vs. withhold responses to emotional faces (CF, CH)] and valence (happy face blocks (CH, HC) vs. fearful face blocks (CF, FC) and their interaction as within-subject factors. The primary effect of interest was the instruction effect, in that it indicates whether emotional faces facilitate correct responding (greater hit rates) only when they are targets or facilitate error responses (greater FA rates) only when they are non-targets. Because advanced age has been shown to degrade performance on the EGNG task (55) and the CoUD group was significantly older (c.f. Table 1), and because we wished to assess replication of the Tottenham et al. (41) finding in controls, these ANOVA were first performed for each group separately.

After analyzing each group singly, we next further examined main and interactive effects of group on task behavior using repeated-measures ANCOVA, adding group as the between-subject factor, while controlling for age as a covariate. These ANCOVA of group effects were first performed for happy (CH, HC) and fearful (CF, FC) block pairs separately, for illustrative purposes. Finally, in light of findings that among emotional faces, fearful or angry faces may capture attention most strongly (53, 56), we performed omnibus analyses across all four trial types, with valence added as a within-subject factor.

We also wished to determine whether attentional bias toward faces (ostensibly indexed by altered RT) related to motivation and affective symptomatology, as a marker of internal validity. Notably, eye-tracking research indicates that individuals with anxiety show a bias in initial gaze orientation toward fearful faces (26), suggesting increased reflexive attentional capture by threat. To assess this, we related PROMIS Anxiety 4a total scores in each group to Fear Approach Bias (FAB) scores, calculated as the difference between median RT to targets under CF conditions minus median RT to targets under FC conditions. Thus, higher (positive) values indicate bias toward (i.e., faster responding to) fearful faces as targets relative to expressionless faces as targets. Similarly, PROMIS Depression total scores were related to Happy Approach Bias (HAB) scores in each group, calculated as the difference between median RT to expressionless targets (under CH conditions) minus RT to happy targets (under HC conditions).

## Results

In addition to the participants analyzed in this report (c.f. Table 1), other participants (*n* = 7 CoUD, *n* = 8 CaUD, *n* = 17 OUD, *n* = 16 controls) had been screened and tested, but their EGNG performance data indicated that they had instructions reversed in at least one task block (A <0.5), and so were excluded. Due to technical/software malfunction, data were not available on one or more specific task blocks from some participants. These participants were thus not included in those repeated-measures analyses that invoked the missing datum.

### Effects of Instruction, Valence and Their Interaction on EGNG Performance Within Each Group

**Figure 2:**
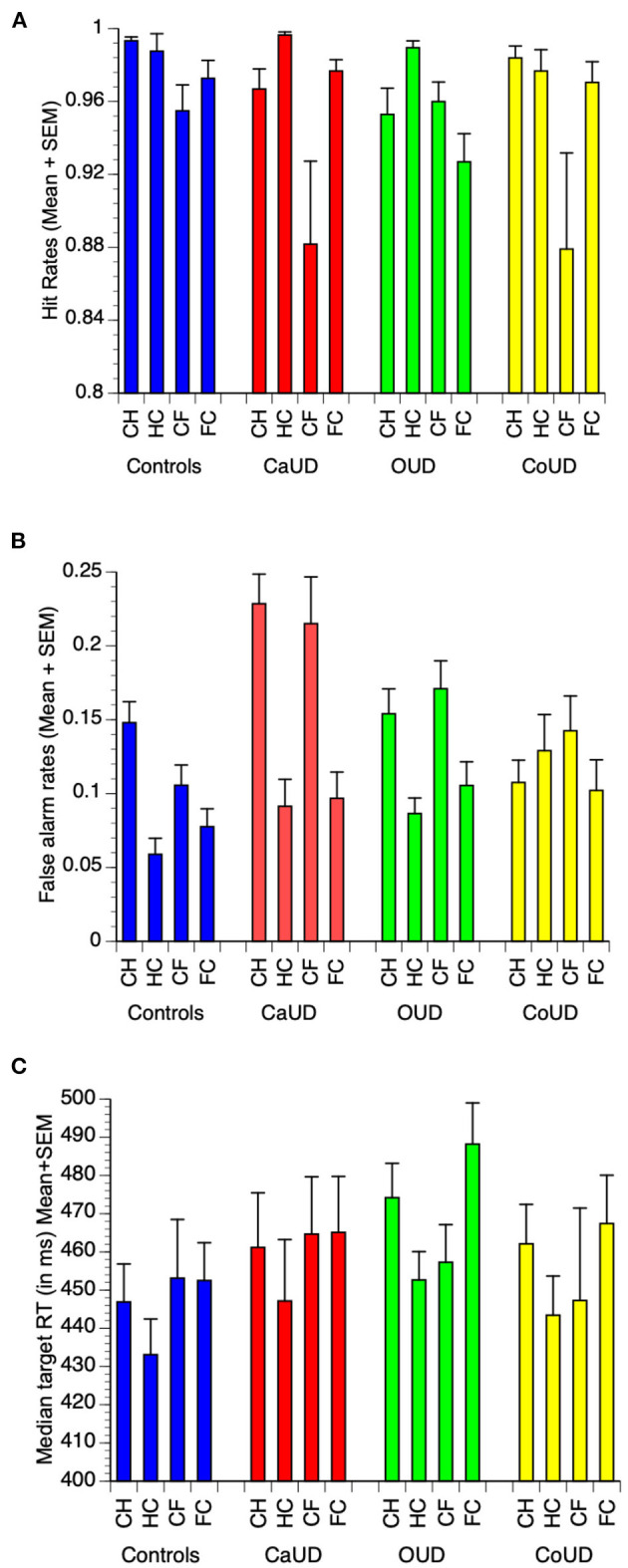
Raw mean (and SEM) values for hit rates **(A)**, false alarm commission error rates **(B)**, and median reaction time (RT) to targets **(C)** in each of controls, cannabis use disorder (CaUD), opioid use disorder (OUD), and cocaine use disorder (CoUD). Trial types: “CH” = respond to calm, withhold to happy; “HC” = respond to happy, withhold to calm; “CF” = respond to calm, withhold to fearful; “FC” = respond to fearful, withhold to calm”.

For brevity, detailed ANOVA statistics (significant main and interactive effects of instruction and valence) are presented in Supplementary Table 1. Raw mean (± SEM) task performance values for the key task metrics are shown in Figure 2. Signal detection parameter results (A and logB) are presented in Supplementary Material.

#### Task Behavior in Control Participants

##### Hit Rates

In controls, there was a main effect of emotion valence, wherein hit rates were higher in happy blocks (HC, CH) relative to fearful blocks (FC, CF) (Figure 2A).

##### False Alarm Rates

There was a main effect of instruction on FA rates, which were significantly higher when emotional faces were non-targets (Figure 2B). A significant instruction X valence interaction effect indicated that the facilitating effect of emotional faces to elicit false alarms was more pronounced for happy faces.

##### Target RT

There were no main effects of instruction or valence, nor any instruction X valence interaction effect on target RT (Figure 2C).

#### Task Behavior in Cannabis Use Disorder Participants

##### Hit Rates

In participants with CaUD, as with controls there was a main effect of valence, wherein hit rates were higher in happy blocks (HC, CH) relative to fearful blocks (FC, CF) (Figure 2A). In addition, CaUD participants also showed a main effect of instruction, wherein hit rates were higher to emotional faces. However, this was not specific to either emotion (no interaction effect).

##### False Alarm Rates

As with controls, there was a main effect of instruction on FA rates, which were significantly higher when emotional faces were non-targets. There were no main or interactive effects of valence.

##### Target RT

There were no main effects of instruction or valence, nor any instruction X valence interaction effect on target RT.

#### Task Behavior in Opioid Use Disorder Participants

##### Hit Rates

In participants with OUD, as with controls there was a main effect of valence, wherein hit rates were higher in happy blocks (HC, CH) relative to fearful blocks (FC, CF) (Figure 2A). Unlike controls and CaUD participants, OUD showed no main effect of instruction on hit rates. OUD participants instead showed an instruction X valence effect wherein hit rates were enhanced by happy faces as targets, but suppressed by fearful faces as targets.

##### False Alarm Rates

As with controls and CaUD participants, FA rates were significantly higher when emotional faces were non-targets, but with no main or interactive effects of valence.

##### Target RT

There were no main effects of instruction or valence on target RT, however, unlike other groups, OUD participant showed a significant instruction X valence interaction effect on target RT that paralleled FA rates, wherein happy faces as targets sped responses, but fearful faces as targets slowed responses.

#### Task Behavior in Cocaine Use Disorder Participants

##### Hit Rates

In participants with CoUD there was a main effect of emotion valence, wherein hit rates were higher in happy blocks (HC, CH) relative to fearful blocks (FC, CF) (Figure 2A). Unlike controls and CaUD groups, CoUD participants did not show a main effect of instruction nor an instruction X valence effect on hit rates.

##### False Alarm Rates

There were no main or interactive effects of instruction or valence on FA rates.

##### Target RT

There were also no main effects of instruction or valence, nor any instruction X valence interaction effect on target RT.

#### Synopsis of Signal Detection Statistics in Each Group Singly

All four groups showed a main effect of instruction on target-catch discriminability (A), where A values were higher when emotional faces were targets. In addition, OUD participants showed both a valence X instruction interaction effect of better discriminability with happy faces as the emotional target (HC > CH) but no difference with fearful faces (FC = CF). These effects are visualized in Supplementary Figure 1A. Most groups also showed significant valence X instruction effects on response bias (LogB; see Supplementary Figure 1B).

### Main and Interactive Effects of Participant Group on EGNG Performance

**Figure 3:**
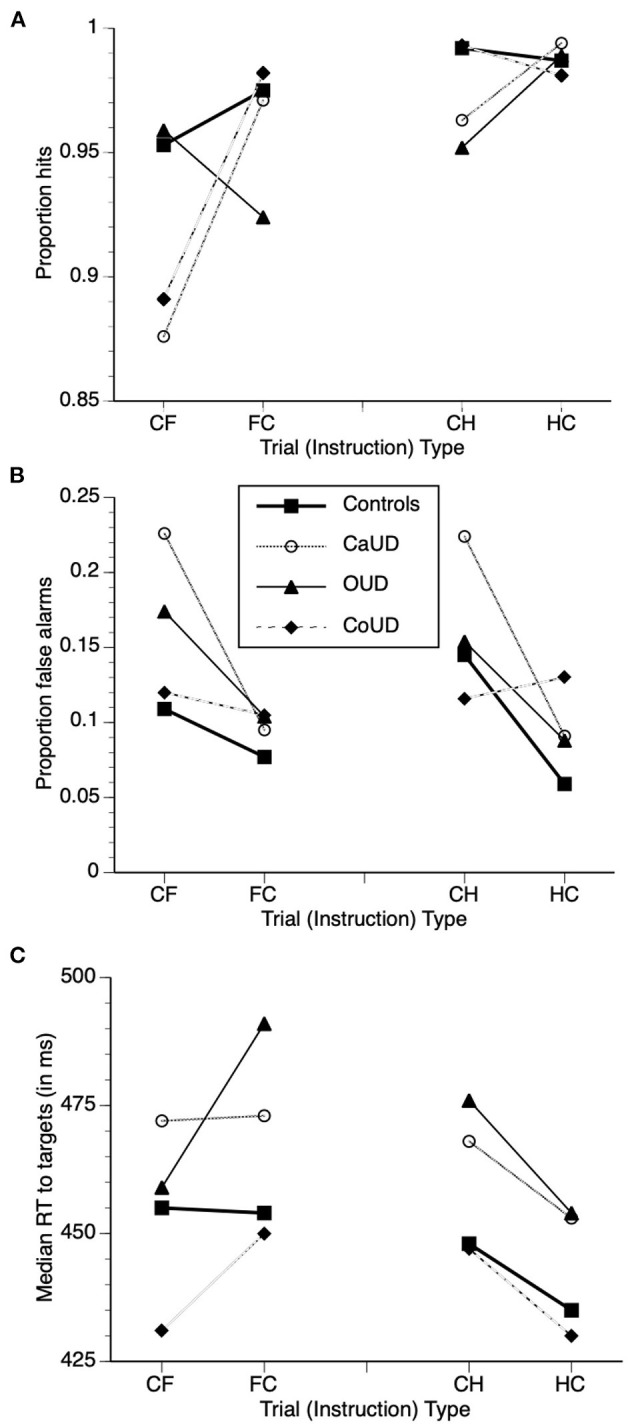
Adjusted mean task performance after controlling for age, with overlaid adjusted means of hits **(A)**, false alarm commission errors **(B)**, and median reaction time (RT) to targets **(C)** in the four participant groups. Trial types: “CH” = respond to calm, withhold to happy; “HC” = respond to happy, withhold to calm; “CF” = respond to calm, withhold to fearful; “FC” = respond to fearful, withhold to calm”.

Adjusted mean values of task performance parameters (after controlling for age) in the four groups are also juxtaposed in Figure 3 to illustrate groupwise behavior patterns that drove the repeated-measures ANCOVA results that follow. Specific statistics of each ANCOVA are presented in Table 2 instead of enumerated in text for brevity. Main effects and interaction effects of subject group, valence and instruction set on signal detection theory statistics A (sensitivity) and LogB (response bias) were also calculated and are described and presented in Supplementary Material.

**Table 2:** Repeated-measures analysis of covariance (ANCOVA) results^*^.

**Happy face blocks (CH, HC)**	**df hypoth**	**df error**	***F***	***P***
**Hit rates**				
Group	3	194	1.367	0.254
Instruction	1	194	0.885	0.348
Group × instruction	3	194	4.474	**0.005**
**False alarm rates**				
Group	3	193	2.614	0.053
Instruction	1	193	6.435	**0.012**
Group × instruction	3	193	5.368	**0.001**
**Median target RT**				
Group	3	194	1.892	0.132
Instruction	1	194	0.227	0.634
Group × instruction	3	194	0.391	0.760
**Fearful face blocks (CF, FC) hit rates**				
Group	3	190	1.051	0.371
Instruction	1	190	0.510	0.476
Group × instruction	3	190	5.583	**0.001**
**False alarm rates**				
Group	3	193	3.129	**0.027**
Instruction	1	193	2.063	0.153
Group × instruction	3	193	3.972	**0.009**
**Median target RT**				
Group	3	193	1.617	0.187
Instruction	1	193	0.048	0.826
Group × instruction	3	193	1.422	0.238
**All blocks (CH, HC, CF, FC) hit rates**				
Group	3	190	1.161	0.326
Instruction	1	190	0.287	0.593
Valence	1	190	0.208	0.649
Group × instruction	3	190	4.055	**0.008**
Valence × instruction	1	190	0.568	0.452
Group × valence	3	190	1.033	0.379
Group × valence × instruction	3	190	6.410	0.001
**False alarm rates**				
Group	3	190	3.622	**0.014**
Instruction	1	190	0.393	0.532
Valence	1	190	4.760	0.30
Group × instruction	3	190	7.039	**0.001**
Valence × Instruction	1	190	8.584	**0.004**
Group × valence	3	190	0.952	0.416
Group × valence × instruction	3	190	1.522	0.210
**Median target RT**				
Group	3	193	1.905	0.130
Instruction	1	193	0.000	0.996
Valence	1	193	0.004	0.950
Group × instruction	3	193	0.725	0.538
Valence × instruction	1	193	0.153	0.696
Group × valence	3	193	0.258	0.856
Group × valence × instruction	3	193	1.736	0.161

* *Age included in all models as covariate. Bold denotes P < 0.05 (statistical significance)*.

### Effects of Happy Faces on Behavior (Across CH and HC Trial Types)

#### Hit Rates

Across all four participant groups, there were no main effects of group or instruction on hit rates across CH and HC blocks (see Table 2 for ANCOVA statistics). The group X instruction interaction, however was significant, wherein OUD participants showed reduced hit rates relative to controls under CH conditions (c.f. Table 1, right), but this group difference was not evident under the HC instruction [i.e., when happy faces were targets (Figure 3)].

#### False Alarm Rates

There was no significant main effect of group on false alarm rates across all participants. There was a main effect of instruction, such that false alarm rates were significantly higher when happy faces were non-targets relative to when they were targets. There was a significant group X instruction interaction effect, however, wherein the instruction effect was more pronounced in CaUD participants but minimal in CoUD participants.

#### Reaction Times

There were no main effects of group, instruction, or group X instruction interaction on RT.

### Effects of Fearful Faces on Behavior (Across CF and FC Trial Types)

#### Hit Rates

Across all four participant groups, there were no main effects of group or instruction on hit rates across CF and FC blocks (see Table 2 for ANCOVA statistics). The group X instruction interaction, however was significant, wherein hit rates were higher when fearful faces were targets in CaUD and CoUD participants vs. when they were non-targets. In contrast, this instruction effect was minimal in controls, and reversed in OUD participants, who showed lower hit rates to fearful faces as targets vs. non-targets (Figure 3).

#### False Alarm Rates

There was no main effect of instruction. There was a significant main effect of group on false alarm rates in CF and FC blocks across all participants, which was driven by greater overall errors in CaUD and to a lesser extent OUD participants relative to controls. There was a significant group X instruction interaction effect, however, wherein higher error rates when fearful faces were non-targets (CF) relative to when fearful faces were targets (FC) occurred specifically in CaUD and OUD participants (Figure 3).

#### Reaction Times

There were no main effects of group, instruction, or group X instruction interaction on RT.

### Task Behavior Across All Trial Types (Both Valences Combined)

In the concluding analyses below, all four task conditions were admixed in the same model, to directly assess main and interactive effects of the valence (positive vs. negative) of emotional faces on behavior. ANCOVA statistics are also presented in Table 2.

#### Hit Rates

There were no main effects of group, instruction or face image valence on hit rates. There was however, an instruction X group interaction effect on hits. Whereas, controls showed no appreciable effect on instruction on hits, CaUD participants showed decreased hit rates when expressionless faces were targets (Figure 2). Further, there was a significant instruction X group X valence interaction effect. In the OUD group (only), hit rates were higher when happy faces were targets than when happy faces were non-targets, whereas hit rates were lower when fearful faces were targets than when fearful faces were the non-targets.

#### False Alarm Rates

There were main effects of valence, group, and instruction, wherein false alarms were higher when emotional faces were non-targets (across all groups) and were lower in controls compared to the SUD groups (across both valences and instruction sets) (Figure 2). A significant instruction X valence effect indicated that the effect of happy faces as non-targets to elicit false alarm responses was present in more participant groups (including controls) vs. fearful faces as non-targets (which did not elicit this effect in controls). Finally, a significant group X instruction interaction effect indicated that an instruction effect (emotional faces as non-targets eliciting more false alarms) was specific to CaUD and OUD groups (across both valences. The valence X instruction X group interaction was not significant.

#### Reaction Times

There were no significant main or interaction effects of group, valence, or instruction on (median) reaction times to targets.

### Relationships Between Attentional Bias and Affective Symptomatology

In multiple regression across all participants, PROMIS Anxiety 4a total scores showed independent positive relationships with FAB scores (Beta = 0.316, *P* < 0.001), wherein responses to fearful faces were faster in participants with more anxiety irrespective of (i.e. controlling for) participant group. Anxiety 4a scores also showed a relationship to SUD diagnosis (*F*_(6, 182)_ = 5.284, *P* = 0.018 (rank order of adjusted means: OUD > CoUD > CaUD > control) after controlling for each for age and sex (see Supplementary Figure 2). PROMIS Depression 4a total scores, however, did not show a similar relationship with HAB scores (as a potential marker for attentional capture by happy faces) (Beta = 0.060, *P* = 0.412).

## Discussion

We administered an emotional go-nogo task to neurotypical controls and to individuals with disordered use of different substances, in order to determine if individuals with different SUDs show aberrations in early SIP compared to controls. We found that emotional face targets elicited higher hit rates in all groups, with the exception of reduced hits to fearful faces as targets in participants with OUD. Relatedly, this increased attentional capture by emotional faces as non-targets elicited more “false alarm” commission errors, especially in CaUD participants.

Our finding that emotion-conveying faces generally elicited more responsiveness than emotionless faces replicates findings of the developmental comparison study that refined this task (41) and also the findings of a recent comparison between older and younger adults (55), wherein signal-detection statistics were improved when emotional faces were targets. This directionality also reflects the cognitive ease of reporting negative (57) or happy faces (58) among arrays of faces in visual search tasks. That emotion-laden faces are more salient is thought to be evolutionarily advantageous in that signals for strong emotion (especially anger and fear) could prompt survival fight or flight responses, whereas signals of happiness could prompt cooperative or affiliative behavior (59, 60). This salience is also evident during fMRI, where emotional faces typically activate visual cortex (fusiform area) and salience network (insula, anterior cingulate cortex) more than expressionless faces (61). Here in the context of a go-nogo task, this salience had the combined effects of increasing hit rates to emotional faces as targets, and increasing commission errors to emotional faces as non-targets. There was a tendency toward faster RT to emotional faces as well in most groups, but this was not significant due to between-subject variability.

Among the participant groups, CaUD participants showed the highest commission error rates when emotional faces were non-targets (especially withholding responses to happy faces) relative to when emotional faces were targets. This motoric impulsivity in CaUD participants stands in contrast with previous findings in more typical go-nogo or stop signal tasks, wherein cannabis users or persons with CaUD are nearly unique among substance users/abusers in NOT showing increased motoric impulsivity compared to controls (40). This impulsivity in CaUD was also evident amid similar fluid intelligence estimates across all study groups, suggesting some specificity of an SIP abnormality in CaUD. One account for this unique impulsivity with social stimuli may lie in cross-sectional personality studies, wherein persons with CaUD show higher scores of openness (to new experiences) (33, 62, 63) and novelty-seeking (32). In light of how happy faces interfere with aspects of fear conditioning by signaling *safety* (64), it stands to reason that novel safety signals would elicit approach behavior more readily in individuals especially open to novel experiences. We note, however, that CaUD individuals also have lower agreeableness in some reports (62), especially when they are comorbid tobacco smokers (65). Future studies of SIP in SUD could include personality assessments as potential mediators of SIP biases.

In our omnibus analyses of all groups there was a significant instruction X valence effect, where the instruction effect (emotion as target vs. emotion as non-target) was more evident with happy faces than with fearful faces, as evidenced in FA rates. This underscores the intuitive motivational appeal of happy images. As noted above, this effect was especially pronounced in CaUD participants. While speculative, this approach bias may suggest that persons who abuse cannabis may be especially sensitive to positively-valenced social cues, such that early attentional processing of such positive cues may interfere with inhibitory motoric processes. Under this conceptualization, persons with CaUD could be seen as strongly motivated toward exuberant and novel social experiences relative to non-drug users and persons with other SUDs. Further investigation of this CaUD-specific effect could investigate early neural attentional signatures in response to social reward. In addition, given that motivation for social reward is thought to be generally attenuated in SUDs, the possibility that persons with CaUD possess heightened intrinsic motivation toward positive social cues could be leveraged during treatment, such as by cognitive restructuring and behavior modification aimed at increasing exposure to non-cannabis-related rewarding social activities and facilitating reinforcement of prosocial behavior.

Based on several findings that stimulant use disorders are linked to more motoric impulsivity and other executive function problems than other SUD (40, 66), we anticipated that CoUD participants would show the most FA commission errors, and possibly the greatest effect of social stimuli to elicit more FA. However, participants with CoUD were somewhat unique in that they did *not* show the differential effect of task instruction on FA rates (see Figure 2B) observed in other groups. Moreover, their age-adjusted residual mean FA rates generally were not greater than those of other groups (see Figure 3B).

One possibility for these findings may be a deficit in detection of the emotional information (i.e., valence) of the faces themselves in CoUD participants. While this deficit has also been found in each of CaUD (14, 15) and OUD (16), the deficit may perhaps be more severe in CoUD (12, 13). However, the CoUD participants herein, who evidenced task comprehension sufficient to be included in this analysis, still showed performance suggestive of adequate recognition of emotion itself. Rather, it appears that CoUD participants are characterized by reduced *bias toward* social information, akin to controls. One possibility is simply that these CoUD participants exerted better self-control than CaUD and OUD participants. An alternative possibility that assumes reduced executive function in CoUD may be that CoUD participants (especially the significantly older ones in our sample) may have a higher stimulus duration threshold for full semantic processing of emotional faces than other groups—a threshold that was not as often met with the brief duration (500 ms) of presentations of facial images. It may be that the “gist” of the emotion based on simple stimulus features (e.g., proportion of white in eyes in fear) may have been sufficiently encoded and reconciled with online maintenance of task goals in the CoUD participants, but where the semantic emotional (or theory-of-mind) meaning of the faces was not encoded with sufficient rigor to induce a cognitive approach bias. Alternatively, emotional faces may simply not have as robust a motivational significance (positive or negative) in CoUD even if semantically encoded. Future research designs might parameterize task difficulty and detectability of emotion (e.g., facial “morphs”) to investigate this in more detail, or could collect more collateral social functioning information.

The other idiosyncratic finding regarding the salience of emotional faces was the increased attentional capture by fearful faces in OUD participants, as indicated by slower response times to fearful targets and reduced correct responses to fearful faces as targets that was reversed with regard to happy faces. Similar slower processing to anger stimuli has been found in psychopathy (67), interpersonal aspects of which are more pronounced in OUD relative to stimulant use disorder (35). Assuming fearful images convey threat, this finding stands in contrast to the reduced loss aversion in individuals with OUD on affective decision-making tasks (68). However, this finding is concordant with other data suggestive of increased mood abnormalities and emotional/affective psychopathic traits in OUD compared to other SUD (35).

Relatedly, across all participants, each of SUD group and attentional bias toward fearful face targets (faster RT relative to expressionless face targets) independently and positively correlated with anxiety symptomatology after controlling for age and sex. The independent correlation across all participants between self-reported anxiety and the net bias toward fearful faces (speeding of responses to fearful face targets relative to expressionless targets) reflects findings of implicit (attentional) bias toward fearful faces in the anxiety literature (26), where anxious individuals frequently “approach” fearful stimuli (e.g., with eye saccades and initial gaze fixation). Increased incidence of mood symptomatology in OUD has long been documented in comorbidity studies (69), and has also been detected as a more specific feature of OUD relative to stimulant disorder in machine-learning-based analysis of laboratory behavioral and self-report features (35).

This investigation of SIP across several SUDs should be considered in light of several limitations. First, behavior opposite that of instructions in several participants suggested that they did not remember the response contingency (instruction presented at start of a block) in one or more task blocks, and so were excluded from analysis. This may have constrained our sample to persons with SUD with adequate working memory. A new version of the EGNG task has since been programmed for distribution to interested colleagues that features static instructions (e.g., “Respond to calm”) at the bottom screen margin. Second, the EGNG task was not difficult, with A values near 1 (or even exactly 1) in some task blocks in some participants. This likely compressed group differences. Future versions of the task could scale back the duration of stimulus presentation or could present graded “morphs” of faces that are less clear in conveying emotion. Third, a cohort effect (cocaine initiates of the '80 s) resulted in a greater proportion of CoUD participants being older adults, necessitating covariation for age. Fourth, data on treatment status (endorsed by callers in telephone pre-screens) was not retained for this analysis, so generalizability to treatment vs. non-treatment samples cannot be assumed. Finally, as is frequent with American community samples of drug users, polysubstance use was widespread. It seems likely that substance-specific SIP effects would have been more pronounced in “purer” mono-substance samples. However, the goal of the core PhAB study was to test feasibility of assessments in Americans with SUD such as they are.

In conclusion, this experiment explored SIP in several SUDs in the context of a signal-detection-based affective impulsivity task, and revealed that CaUD individuals in particular showed exaggerated approach behavior and poorer inhibition toward non-target happy faces, whereas OUD participants showed greater attentional capture by fearful faces as targets. This sensitivity to social information (especially to happy faces) in most participant groups with SUD could not only provide a mechanism of social reinforcement for cannabis use in CaUD, but also the potential for social reinforcement of SUD recovery generally. Future experiments could add pupillometry or other physiological metrics of arousal to the EGNG task, as well as additional phenotypic assessments of real-world social function or extent of social networks. Finally, larger sample sizes could explore sex differences in SIP aberrations in SUD.

## Data Availability Statement

The raw data supporting the conclusions of this article will be made available by the authors, without undue reservation.

## Ethics Statement

The studies involving human participants were reviewed and approved by the Institutional Review Board (IRB) of Virginia Commonwealth University. The patients/participants provided their written informed consent to participate in this study.

## Author Contributions

JB analyzed the data and drafted the manuscript. LK-M, TR, and FM designed the core study recruitment plan and assessment battery, which included the emotional go-nogo task. DH contributed clinical perspective to the findings. All authors edited the manuscript draft and contributed content to it.

## Conflict of Interest

The authors declare that the research was conducted in the absence of any commercial or financial relationships that could be construed as a potential conflict of interest.
