# Supplementary material for: Social Information Processing in Substance Use Disorders: Insights From an Emotional Go-Nogo Task
Source: Front Psychiatry. 2021 May 28;12:672488. doi: 10.3389/fpsyt.2021.672488 (PMC8193089; doi:10.3389/fpsyt.2021.672488)
Supplement: Supplementary file 1 [file Data_Sheet_1.PDF]

Social information processing in Substance Use Disorders: Insights from an emotional go-nogo task

SUPPLEMENTAL ONLINE DOCUMENT:

Table S1 — Repeated-measures analysis of variance (ANOVA) results

		df hypoth	df error	*F*	*P*
**Controls**					
Hit Rates	Instruction	1	55	0.663	.419
	Valence	1	55	6.531	**.013**
	Instruction X Valence	1	55	1.803	.185
False Alarm rates	Instruction	1	55	26.334	**<.0001**
	Valence	1	55	1.009	.320
	Instruction X Valence	1	55	6.795	**0.012**
Median Target RT	Instruction	1	57	0.871	.355
	Valence	1	57	3.154	0.081
	Instruction X Valence	1	57	0.889	.350
**Cannabis Use Disorder (CaUD)**					
Hit Rates	Instruction	1	30	6.746	**.014**
	Valence	1	30	5.197	**.030**
	Instruction X Valence	1	30	2.220	.147
False Alarm rates	Instruction	1	30	39.013	**<.0001**
	Valence	1	30	0.043	.837
	Instruction X Valence	1	30	0.164	.689
Median Target RT	Instruction	1	30	1.496	.231
	Valence	1	30	1.705	.202
	Instruction X Valence	1	30	2.556	.120
**Cocaine Use Disorder (CoUD)**					
Hit Rates	Instruction	1	30	3.124	.087
	Valence	1	30	4.963	**.034**
	Instruction X Valence	1	30	3.651	.066
False Alarm rates	Instruction	1	30	0.256	.616
	Valence	1	30	0.051	.823
	Instruction X Valence	1	30	3.940	.056
Median Target RT	Instruction	1	30	0.003	.957
	Valence	1	30	0.125	.726
	Instruction X Valence	1	30	2.083	.159
**Opioid Use Disorder (OUD)**					
Hit Rates	Instruction	1	75	0.003	.957
	Valence	1	75	10.874	**.002**
	Instruction X Valence	1	75	9.365	**.003**
False Alarm rates	Instruction	1	75	23.795	**<.0001**
	Valence	1	75	1.312	.256
	Instruction X Valence	1	75	0.000	.999
Median Target RT	Instruction	1	76	0.353	.554
	Valence	1	76	3.147	.080
	Instruction X Valence	1	76	18.689	**<.0001**

Effects of group, valence, instruction and their interaction on signal detection metrics

As secondary task performance metrics of interest to some readers, we also calculated the omnibus signal detection statistics A and b (Zhang and Mueller, 2005). A is a non-parametric statistic of sensitivity (overall performance accuracy) akin to d-prime, but does not require substitution of values in performances with no errors. An A value of 1.0 indicates a perfect task performance. The metric B (log-transformed for normality) reflects response bias, where higher values indicate a more conservative responding strategy, and lower values a more liberal responding strategy. Results of repeated-measures ANOVA on A and logB are as follows:

Sensitivity (A):

There were no main effects of instruction (respond to emotion vs withhold to emotion), valence (fear blocks vs happy blocks) on sensitivity A. However, there was a main effect of participant group ($F(3,188) = 3.745$, $p = .012$) wherein CaUD participants had reduced sensitivity compared to other groups. A significant group X instruction interaction effect ($F(3,188) = 7.093$, $p < .001$) was driven by how CaUD participants (and OUD) participants showed reduced A when emotional faces were non-targets (See Figure S1, part A below). Finally, a significant valence X instruction X group interaction ($F(3,188) = 3.610$, $p = .014$) showed that this instruction effect was apparent in CaUD participants for both happy and fearful non-target faces, but in OUD participants was present for happy faces only.

Response bias (logB):

There were no main effects of instruction (respond to emotion vs withhold to emotion), valence (fear blocks vs happy blocks) or subject group on log-transformed response bias metric logB. A significant valence X instruction interaction effect ($F(1,188) = 3.909$, $p = .049$) was driven by more conservative responding when happy faces were the target (HC block) versus non-target, but this was not as consistent when fearful faces were targets (See Figure S1, part B below). Finally, a significant valence X instruction X group interaction ($F(3,188) = 4.230$, $p = .006$) showed further that the valence X instruction interaction effect was more specific to controls and CoUD participants.

Figure S1

A.

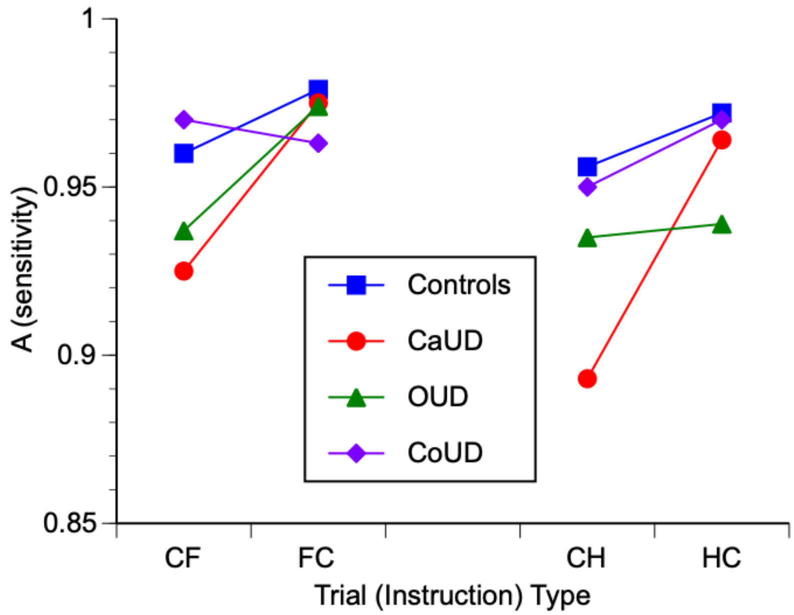

B.

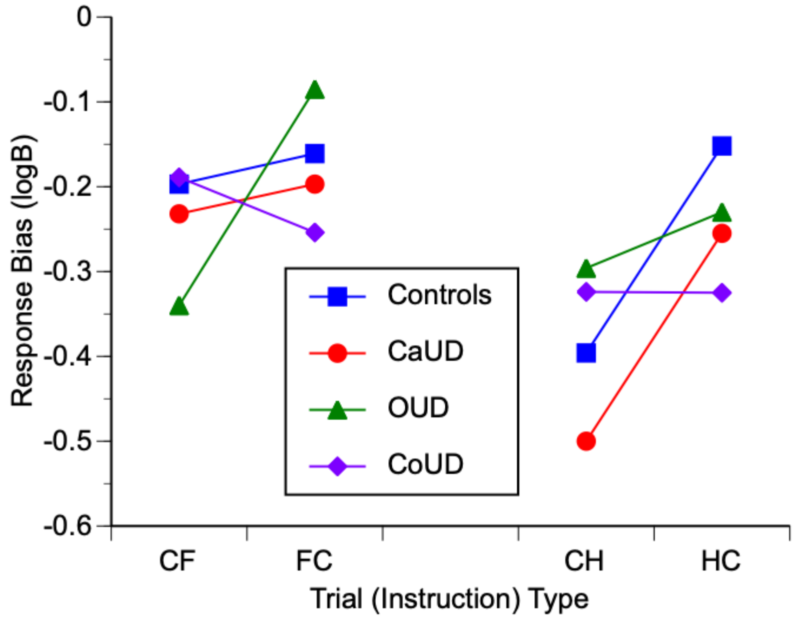

Figure S1 legend: Adjusted mean values for sensitivity (A) (part A) and log-transformed response bias B (part B) as a function of task instruction and emotion valence. This analysis controlled for age as a covariate, due to greater age in CoUD participants.

Relationships between attentional bias and affective symptomatology

In multiple regression, Fear Attentional Bias (FAB) scores showed a positive relationship with PROMIS Anxiety 4a total scores across all participants (Beta = .316, $P < .001$; see Figure S2 below). This indicates that

participants with more anxiety (irrespective of participant group) responded faster to fearful faces (as targets) than to expressionless faces when fearful faces were present as non-targets. The relationship was still significant (Beta = .228, P = .002) after controlling only for age and sex.

Figure S2. Leverage plot of relationship between PROMIS Anxiety scores and FAB scores

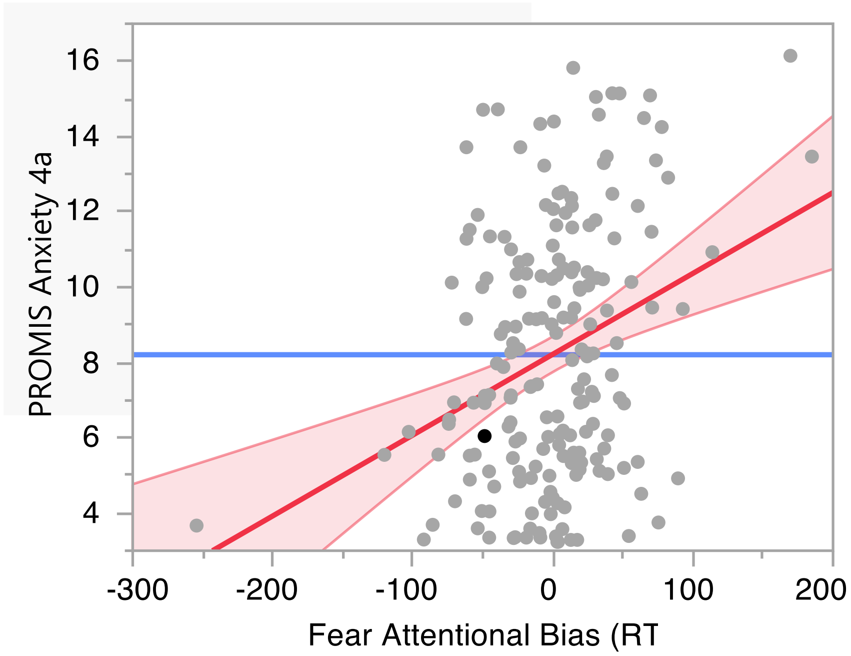

Figure S2 legend: Leverage plot of multiple regression analysis wherein Fear Attentional Bias (FAB) scores were entered into the model along with sex and age, and SUD group as simultaneous covariates, and total scores of the PROMIS Anxiety 4 scale as the dependent variable. The plot illustrates the significant positive relationship between attentional capture by fearful faces (as targets) compared to task blocks when expressionless faces were targets (Beta = .316, $P < .001$).

References for Supplemental Online Document:

Zhang, J., Mueller, S.T., 2005. A note on ROC analysis and non-parametric estimate of sensitivity. Psychometrika 70, 203–212. https://doi.org/10.1007/s11336-003-1119-8
